# On the Use of the Repeated-Sprint Training in Hypoxia in Tennis

**DOI:** 10.3389/fphys.2020.588821

**Published:** 2020-12-18

**Authors:** Cyril Brechbuhl, Franck Brocherie, Sarah J. Willis, Thomas Blokker, Bernard Montalvan, Olivier Girard, Gregoire P. Millet, Laurent Schmitt

**Affiliations:** ^1^French Tennis Federation, Independent Researcher, Paris, France; ^2^Institute of Sports Science, University of Lausanne, Lausanne, Switzerland; ^3^EA7370 Laboratoire Sport, Expertise et Performance, Institut National du Sport, de l’Expertise et de la Performance, Paris, France; ^4^Fédération Française de Tennis (FFT), Paris, France; ^5^Faculty of Science, School of Human Sciences, University of Western Australia, Perth, Australia; ^6^Centre National de Ski Nordique et de Moyenne Montagne (CNSNMM), Prémanon, France

**Keywords:** sport-specific fitness, hypoxia, repeated-sprint, tennis performance, repeated sprint ability, maximal aerobic exercise intensity

## Abstract

**Purpose:**

To examine physiological and technical responses to repeated-sprint training in normobaric hypoxia at ∼3,000 m (RSH, *n* = 11) or in normoxia (RSN, *n* = 11) compared to a control group (CON, *n* = 8) in well-trained tennis players. Participants were 28.8 ± 5.9 years old without any previous experience of training in hypoxia.

**Methods:**

In addition to maintaining their usual training (CON), both RSH and RSN groups completed five tennis specific repeated-shuttle sprint sessions (4 × 5 × ∼8 s maximal sprints with ∼22 s passive recovery and ∼5 min rest between sets) over 12 days. Before (Pre), the week after (Post-1) and 3 weeks after Post-1 (Post-2), physical/technical performance during Test to Exhaustion Specific to Tennis (TEST), repeated-sprint ability (RSA) (8 × ∼20 m shuttle runs—departing every 20 s) and heart rate variability (HRV) were assessed.

**Results:**

From Pre to Post-1 and Post-2, RSH improved TEST time to exhaustion (+18.2 and +17.3%; both *P* < 0.001), while the “onset of blood lactate accumulation” at 4 mmol L^–1^ occurred at later stages (+24.4 and +19.8%, both *P* < 0.01). At the same time points, ball accuracy at 100% V̇O_2m__ax_ increased in RSH only (+38.2%, *P* = 0.003 and +40.9%, *P* = 0.007). Markers of TEST performance did not change for both RSN and CON. Compared to Pre, RSA total time increased significantly at Post-1 and Post-2 (−1.9 and −2.5%, *P* < 0.05) in RSH only and this was accompanied by larger absolute Δ total hemoglobin (+82.5 and +137%, both *P* < 0.001). HRV did not change either supine or standing positions.

**Conclusion:**

Five repeated sprint training sessions in hypoxia using tennis specific shuttle runs improve physiological and technical responses to TEST, RSA, and accompanying muscle perfusion responses in well-trained tennis players.

## Introduction

Elite tennis players possess well-developed technical and tactical skills and high fitness level in order to cope with the physical demands of the game. They need to develop a combination of fitness qualities such as speed, agility, repeated sprint ability (RSA), power as well as high aerobic fitness (Reid and Schneiker, 2008). Optimizing training time remains challenging due to congested tennis competition calendars. Repeated-sprint exercise sessions, with the potential of concomitantly increasing maximal oxygen uptake (V̇O_2m__ax_) and speed/power during tests of RSA, represent effective means for physical performance enhancement in tennis (Bishop et al., 2011). Furthermore, it is believed that exercise training prescription should be as specific as possible to facilitate appropriate physiological adaptations (Reilly et al., 2009).

Although repeated-sprint exercise sessions are most frequently performed in normoxia (RSN), their completion in systemic hypoxia (RSH) is increasingly popular in many sports (e.g., soccer, rugby, cross-country skiing) since it can provide additional performance benefits. A recent meta-analysis indicated that mean power during repeated-sprint exercise is further enhanced with RSH compared to RSN (Brocherie et al., 2017a). Additional benefits of RSH have been reported in many intermittent sports including tennis (Faiss et al., 2013a). For example, single sprint time (−4.5%), RSA total time (−3.1%) and sprint decrement (−16.7%) improved in one rookie tennis player during a stress test 3 weeks after undertaking a RSH intervention (Brechbuhl et al., 2018b). Well-trained tennis players also increased time to exhaustion and onset of blood lactate accumulation at 4 mMol L^–1^ as well as the ball accuracy (+13.8%) immediately after five RSH sessions (Brechbuhl et al., 2018a).

To date, there is still scarce evidence about the acute and long-term responses to an on-court sport-specific RSH session (Brechbuhl et al., 2018b). With physical performance only acutely (within days) assessed after the RSH intervention, the long-term (few weeks) maintenance of its effects (if any) is currently unclear (Puype et al., 2013). To our knowledge it has never been investigated with ball hitting; so, a new adapted design is required to assess RSA for tennis players. Particularly, sprinting back and forth, as shown by Gatterer et al. (2015) in soccer players (but without ball hitting), would appear more appropriate than straight line used in previous studies with tennis players.

It was proposed that the effectiveness of RSH comes from an improved muscle blood perfusion, which in turn would benefit from optimized oxygen extraction by fast-twitch fibers (Faiss et al., 2013b). Since a previous study (Brechbuhl et al., 2018a) has reported that RSH can maintain a high level of accuracy at high levels of playing intensity, it would be of interest to investigate if cerebral as well as muscular blood flow could be increased using near infrared spectroscopy (NIRS).

Beyond the effects of RSH on physiological and performance parameters, the impact of this training method on the fatigue state has only been investigated acutely after one session in elite badminton players (Valenzuela et al., 2019). Heart rate variability (HRV) has been presented as a promising tool to differentiate fatigue states, and many studies have reported the influence of the training components on HRV due to a modulation in autonomic nervous system activity (Schmitt et al., 2006). One could speculate that RSH may lead to specific modifications of the neuro-vegetative system activity; e.g., an increase in sympathetic activity combined with a decrease in parasympathetic influence which may lead to a more prolonged state of fatigue (Lehmann et al., 1998). Ramos-Campo et al. (2017) found no differences in autonomic modulation after a high-intensity training session performed under hypoxic or normoxic conditions, but they did not measure the effects on the following days.

Using a double-blind controlled design, the present study aimed to investigate the immediate and prolonged effects of RSH vs. RSN using on-court tennis displacements, compared to control group (CON) on physical and technical tennis performance in well-trained players. We hypothesized that RSH would provide greater gains on RSA-related parameters than RSN as well as more favorable muscle oxygenation levels. We also postulated that RSH would induce a better resistance to fatigue in tennis-specific incremental test, observable mainly near exhaustion, concomitant with enhanced brain, and muscle perfusion measured during RSA and higher sympathetic activity in supine position.

## Materials and Methods

### Subjects

*Thirty-six* competitive tennis players (27 males and 9 females) volunteered to participate in the study. They were all well-trained [i.e., international tennis number (ITN): 1 (elite) to 2 (advanced player)], allowing them to take part in a qualifying draw of International Tennis Federation (ITF) events (*n* = 24); some players were holding a professional (ATP) ranking (*n* = 3). Participants were first contacted by email to inform them of the project. Four of them declined after the medical examinations because they were running out of time or got ankle sprain just before the beginning of the physical testing. The group assignment was finalized during this period of inclusion by the main investigator. Our intention was also to match tennis level and age of our participants between the three groups (cf. Table 1). The sample size of our control group was smaller since these individuals likely had a lower risk of sustaining a musculoskeletal injury compared to the two experimental groups performing repeated “all out” efforts. Two participants had to stop during the training period; one got a muscle injury and one felt a heart arrhythmia. Data of these individuals have been removed from final analysis. *A priori* power analysis using G^∗^Power software (version 3.1.9.3) was conducted to determine the appropriate sample size. Based on the data from a previous study (Brechbuhl et al., 2018a) on effects of repeated-sprints in hypoxia (∼3000 m) vs. normoxia, 27 participants overall were required to yield the targeted analysis power of β = 0.8 at α = 0.05 for three groups and three measurements. To reach the appropriate sample size with potential risks of drop-out or injuries, the present sample was increased to *n* = 36.

**TABLE 1:** Subjects characteristics.

	**RSH (*n* = 11)**	**RSN (*n* = 11)**	**Control (*n* = 8)**
Age (year)	25.7 ± 6.8 [21.1–30.3]	31.3 ± 4.5 [27.0–32.6]	29.6 ± 5.1 [25.3–33.9]
Height (cm)**	179 ± 5 [176–182]	175 ± 7 [169–179]	185 ± 5** [181–189]
Weight (kg)	72.6 ± 8.9 [66.6–78.6]	69.5 ± 8.7 [63.7–75.3]	76.0 ± 12.5 [65.5–86.5]
V̇O_2m__ax_ (mL min^–1^ kg^–1^)	57.2 ± 7.9 [52.0–62.4]	57.8 ± 5.6 [54.0–61.6]	56.7 ± 4.7 [51.4–61.0]
TTE (s)	549 ± 101 [481–617]	621 ± 113 [527–716]	582 ± 72 [540–635]
RSA_TT_ (s)	31.6 ± 1.1 [30.7–32.5]	32.5 ± 2.0 [30.8–34.2]	32.3 ± 2.1 [30.5–34.1]
National tennis ranking (a.u.)	1.9 ± 2.5 [0.2–3.6]	1.5 ± 3.2 [−0.8 to 3.9]	1.0 ± 2.9 [−1.4 to 3.4]

*Values are mean ± SD [95% CI]. Significant differences in the mean values among the groups. ^∗∗^P < 0.01 for Control players taller than those in the RSN group, in bold. The equivalent national ranking [arbitrary units (a.u.)] is ranged from [−5] to [+19]; lower is the ranking, higher is the level of performance.*

The information was disseminated to a list of players with the help of tennis coaches living in Paris and the surrounding region. Inclusion criteria were: (i) age between 18 and 35 years old; (ii) a ranking allowing players to take part in a national team championship; and (iii) no previous exposure to normobaric hypoxia in the past 6 months. Exclusion criteria were any history of altitude-related sickness or health risks that would have compromised the participant’s safety during the experiment. They all have been screened by a sport cardiologist using echocardiography and blood testing. Participants gave their written informed consent after having been fully informed about the experimental procedure. The study was approved by the local ethical committees (French National Conference of Research Ethics Committees—Ile de France 6, No. 77-17) that was performed in accordance with the ethical standards reported (Harriss et al., 2017) and conformed to the recommendations of the Declaration of Helsinki.

### Study Design

According to their competitive tennis level (known from their national ranking), gender and fitness level, participants were split into three groups: RSH (*n* = 12; 9 males and 3 females), RSN (*n* = 12; 8 males and 4 females), and CON (*n* = 8; 7 males and 1 female) (Table 1). Players in CON group continued their regular training routine throughout study. All participants in RSN and RSH groups undertook five additional training sessions across 12 days. Specifically, training sessions occurred on days 1, 3, 5, 9, and 11 for RSH and days 2, 4, 8, 10, and 12 for RSN. Physical and technical evaluations were performed for all tested players before (Pre-), immediately after (Post-1) and 3 weeks (Post-2) after Post-1. The time of day for testing was controlled and matched both within and between groups.

### Specific Training Sessions

**FIGURE 1:**
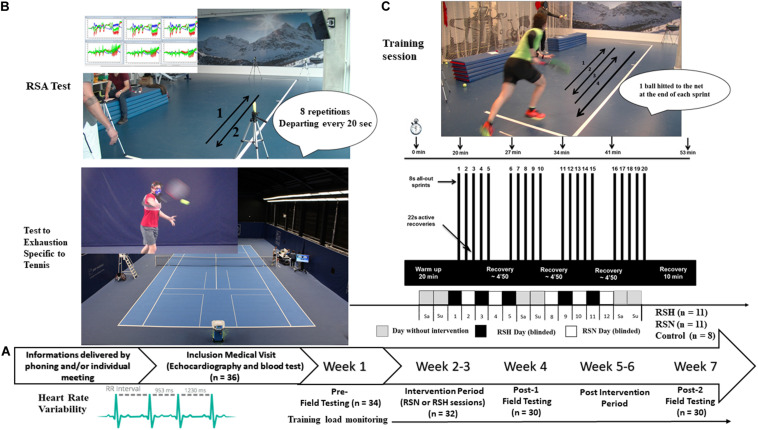
Protocol overview. General procedure **(A)**, and description of a typical repeated-sprint training sessions, in normoxia (RSN) or hypoxia (RSH) **(C)**. Field testing including repeated-sprint ability (RSA), Test to Exhaustion Specific to Tennis, heart rate variability **(B)**, before the training intervention period (Pre-), during the week after (Post-1), and Post-2, 3 weeks after termination of Post-1.

All specific training sessions were performed in an air-conditioned (21°C) normobaric hypoxic room (size 15.04 m × 8.54 m; b-Cat^®^, Netherlands). For RSH, the inspired fraction of oxygen (FiO_2_) was set at 14.5%, equivalent to a simulated altitude of ∼3,000 m. Each session lasted ∼60 min, including a 20 min warm-up phase, the repeated-sprint training routine, and a 10 min cooling-down phase (i.e., a total of ∼300 min for the five sessions during the 12 days training period). Specifically, the repeated-sprint training routine included four sets of four maximal shuttle-run sprints of ∼8 s [i.e., a sequence of two back and forth sprints that was concluded by hitting a ball to a retaining net] interspersed with ∼22 s of recovery (departing every 30 s) (Figure 1). Balls were thrown manually by an investigator (the same during all the investigation) toward a target located on the floor at the time when the preceding ball was hit by the player (cf. Figure 1). Between each set, subjects were allowed a 4 min and 50 s passive rest period. Overall the subjects performed a total of 100 shuttle-runs during the 12 days intervention. A water fountain was on site to facilitate hydration during the resting periods.

Training loads [arbitrary units (a.u.)] were calculated for all subjects as the product of the total session duration (min) and RPE (Foster, 1998) for all training sessions during the experimental protocol including repeated sprints.

### Blinding

This research was conducted in a double-blind, controlled manner. The blinding was carefully controlled (e.g., groups assignment, all materials’ screens covered by a black tissue). For careful blinding purpose (see below), the hypoxic system was also running (FiO_2_ 20.9% equivalent to ∼200 m) for RSN sessions in order to create similar background noise. The efficacy of the blinding process was evaluated upon experiment termination (i.e., immediately after Post-2) by administering Likert scales (100 m marks from 0 to 4,000 m), where each participant had to indicate (separately) which simulated altitude he/she believed he/she had been training in.

### Pre, Post-1, and Post-2 Testing Sessions

A testing session lasted 2.5 h. Participants were invited to arrive at the testing venue 30 min before their scheduled time for repeated sprint training session to test their HRV. One hour after the beginning of the sprints sessions, they completed on the technological court available at the French Tennis Federation a specific test with ball hitting (Brechbuhl et al., 2016a). We describe tests following the order used during the three measurement time points.

### HRV Analysis

HRV was analyzed at arrival on site the day of tests. A detailed description of the protocol is available elsewhere (Schmitt et al., 2013). Briefly, the test lasted 14 min starting with 8 min in the supine position, followed by 6 min in the standing position. The periods analyzed were 3–8 min and 9–14 min, respectively, and were identical for all the subjects and all the tests. Measurement of the interval duration between two R waves of the cardiac electrical activity were performed using a Polar S810 heart rate (HR) monitor (Polar^®^, Kempele, Finland), which has been validated by comparison with ECG measurements (Kingsley et al., 2005). The root mean square differences of successive heart beat intervals (RMSSD) was calculated as a measure of parasympathetic modulation (Task Force of the European Society of Cardiology and North American Society of Pacing and Electrophysiology, 1996). Then the spectral power was calculated with Fast Fourier Transform (FFT) by a specific software (Nevrokard^®^ HRV, Medistar, Ljubljana, Slovenia). The spectral power was measured by frequency bands in ms^2^ Hz^–1^:

0.05–0.15 Hz: low frequency (LF) reflecting predominantly the sympathetic system influence and in relationship with the arterial blood pressure.

0.15–0.40 Hz: high frequency (HF) reflecting the parasympathetic system influence and in relationship with the respiratory rate.

#### Repeated Sprints

The RSA test consisted of eight shuttle-run sprints (Figure 1) departing every 20 s, with passive recovery between efforts [adapted from a previous running test that has been shown to be reliable and valid in estimating RSA (Buchheit et al., 2010)]. Each sprint was initiated from a standing position, 50 cm behind the photocell gate, which started a digital timer. Sprint times were measured to the nearest 0.01 s using photocells connected to an electronic timer (Witty, Microgate^®^, Bolzano, Italy), whose height was adjusted according to the height of the subject’s hip. During the first sprint, subjects were required to achieve at least 95% of their criterion score (i.e., defined as the best of three single back and forth sprints with 2 min of recovery) as a check of any pacing strategy. This measure was realized at the end of a warm-up. All of the subjects satisfied this criterion score. Three seconds before the start of each point, participants were asked to assume the ready position and to await the start signal with a countdown (“3, 2, 1, go”). Three scores were calculated from the RSA test: best sprint time (RSA_best_), total sprint time (RSA_TT_) and percentage of sprint decrement (S_dec_) determined as [RSA_TT_/(RSA_best_ × 8) −1] × 100 (Girard et al., 2011). Maximal lactate concentration (La)_max_ was measured 3 min after the last sprint.

#### Specific Aerobic Capacity

After 40 min of rest, subjects performed an incremental field test up to exhaustion [i.e., the so-called test to exhaustion specific to tennis (TEST)] as detailed previously (Brechbuhl et al., 2016a, 2018a). Briefly, TEST consisted of hitting balls thrown by a “Hightof”^®^ ball machine at constant velocity. Both accuracy and reliability of the machine have been previously reported and appears relevant for field testing and training purposes (Brechbuhl et al., 2016b). Subjects had to hit balls cross-court in a prescribed pattern (i.e., topspin drive), while the landing point for thrown balls was set 3 m in front of baseline (Brechbuhl et al., 2016a). The first TEST stage begun with a ball frequency (BF) of 10 shots min^–1^, thereafter increased by +2 shots min^–1^ every minute until the stage corresponding to a BF of 22 shots min^–1^. From there, increment in BF was set at +1 shots min^–1^ until exhaustion (Brechbuhl et al., 2016a). After each 1 min stage, a 30 s passive recovery break (quiet standing) was implemented.

### Evaluation of Groundstroke Performance

During TEST, groundstroke production was assessed by the mean of two “primary” variables: ball velocity (BV) and ball accuracy (BA). BV (km h^–1^) was measured with the Playsight^®^ system (Israël), which has been approved by the ITF as a tennis player analysis technology for all ITF-sanctioned tournaments. All shots that were hit out, into the net, and to the wrong spot on the tennis court were excluded. BA (%) was defined as the percentage of correct hits in the defined zones. For each stage, BV and BA data were averaged. Finally, because BV_mean_ and BA_mean_ better reflect the overall stroke precision in tennis when combined, a TP_mean_ index was calculated as the product of these two variables. Calculation of overall mean values was based on the values obtained for each stage. We considered technical parameters at 100% V̇O_2m__ax_ with at least 20 balls hit during the stage (BV_max_, BA_max_, TP_max_).

### Physiological Measurements

During TEST, HR (Suunto Ambit2^®^, Vantaa, Finland) and expired air were analyzed continuously (breath-by-breath measurements) for oxygen consumption (V̇O_2_) using a portable gaz analyzer (Metamax II CPX system, Cortex^®^, Leipzig, Germany). Gas and volume calibration of the measurement device were performed before each test according to manufacturer’ instructions. Furthermore, 25 μl capillary blood samples were taken from fingertip and analyzed for (La) (LT-1710, Arkray^®^, Japan) at baseline, during TEST (i.e., during the 30 s recovery periods after every stage until a values of 4 mmol L^–1^ was obtained and thereafter every second stages) and 15 s after exhaustion. V̇O_2m__ax_ was determined by the observation of a “plateau” or leveling off in V̇O_2_ or when the increase in two successive periods was less than 150 mL min^–1^ (Wasserman et al., 2005). The maximal lactate concentration (La)_max_ were also recorded at the end of TEST.

Detection of ventilatory thresholds (VTs) was achieved by identifying points of breakdown in linearity based on ventilatory parameters. The first ventilatory threshold (VT1) was determined using the criteria of an increase in the ventilatory equivalent for oxygen (V̇E/V̇O_2_) with no increase in the ventilatory equivalent for carbon dioxide (V̇E/V̇CO_2_) and departure from the linearity of V̇E caused by a more rapid increase in ventilation. VT2 corresponded to an increase in both V̇E/V̇O_2_ and V̇E/V̇CO_2_ (Wasserman et al., 2005). All VTs assessments were made by visual inspection of graphs of time plotted against each relevant respiratory variable measured during testing. All visual inspections were carried out by two experienced exercise physiologists. The results were then compared and averaged. The difference in the individual determinations of VT2 was < 3%.

The “onset of blood lactate accumulation” (OBLA), defined as the exercise intensity corresponding to 4 mmol L^–1^ blood lactate concentration was also determined. By plotting each subject’s blood lactate concentration against time of TEST completion and visually connecting the data points, we estimated the time to attain OBLA. This physiological variable has been shown to be a good predictor of endurance performance (Bentley et al., 2007).

### Near-Infrared Spectroscopy Measurements

Tissue oxygenation was evaluated using the NIRS technique as described previously by Boushel and Piantadosi (2000). The PortaMon and PortaLite devices (Artinis^®^, Zetten, Netherlands) were used during the RSA test to measure muscle oxygenation of the *vastus lateralis* (PortaMon) and of the prefrontal cortex (PortaLite) at wavelengths between 760 and 850 nm, respectively. All devices were placed into a tight transparent plastic wrap to avoid humidity and create a waterproof barrier for proper function and signal quality. The PortaMon was placed on the lower third of the *vastus lateralis* and attached with double sided tape, then wrapped with tension against the leg to reduce movement during exercise. The position was marked with a permanent pen and images were taken to reproduce the placement in subsequent visits. The PortaLite was attached on the surface of the left prefrontal cortex with double sided tape, then the subject was fitted with a head wrap to create a dark environment and maintain a stable position of the probe. Measurements included a standard differential pathlength factor of 4.0 for the *vastus lateralis* as there is a lack of any clear standard value for the quadriceps (Faiss et al., 2013b) and 6.0 for the prefrontal cortex, similar to Amann et al. (2007). All signals were recorded at the maximum frequency for each device (10 Hz for PortaMon and 50 Hz for PortaLite) and then exported at 10 Hz for further analysis (Oxysoft 3.0.53, Artinis, Netherlands). For analysis, a 4th-order low-pass zero-phase Butterworth filter (cutoff frequency 0.2 Hz) was implemented to reduce artifacts and smooth perturbations in the signal from pedal strokes. All data (except absolute TSI) was normalized to the last 30 s of the seated resting baseline. Because concentrations for deoxyhemoglobin (HHb) values were proposed to be less sensitive to blood flow variations than oxyhemoglobin (O_2_Hb) and changes in O_2_Hb signals might be confounded by rapid blood volume changes during sprints (Buchheit et al., 2009), only (HHb) and total hemoglobin/myoglobin (tHb) were analyzed for relevant interpretations. Differences between maximum and minimum concentrations were defined as the amplitude of the variation for each sprint [Δ (tHb) and Δ (HHb)], and Δ (tHb) was used as an index of blood perfusion (Faiss et al., 2013b). Thus, for example, at the beginning of each sprint, a maximum in (tHb) is observed (i.e., end of each recovery period) and (tHb) decreases to reach a minimum value at the end of each sprint. As well, the absolute maximum tissue saturation index (TSI, %) was obtained from each sprint. This allowed determination of successive sprint and recovery phases to be identified, and sprint phases to be further analyzed. The mean change (Δ) for the eight sprints was defined as the mean difference between maximum and minimum values for the overall repeated sprints series. Mean delta absolute tissue saturation index (Absolute Δ TSI, %), total hemoglobin/myoglobin [Absolute Δ (tHb)], and concentrations of deoxyhemoglobin [Δ (HHb)] were obtained for muscle (from vastus lateralis) and cerebral oxygenation (from prefrontal cortex).

### Statistical Analysis

All data are expressed as the mean ± SD. Mean difference for change between Pre- and Post-1 or Post-2 (expressed as %), and 95% confidence interval (95% CI) were reported when appropriate. One-way ANOVA was used to test differences in training load and initial participants’ characteristics between groups. Two-way repeated measures ANOVA [Time (Pre-, Post-1 vs. Post-2) × Group (RSH, RSN vs. Control)] analysis was applied to compare performance and physiological variables. Pairwise differences were identified using the Tukey *post hoc* analysis procedure adjusted for multiple comparisons. For each two-way repeated measures ANOVA and each pairwise differences, effect sizes were calculated with Cohen’s d (d) with the following criteria: a d of < 0.2 is classified as a trivial, 0.2–0.4 as a small, 0.5–0.7 as a moderate and > 0.8 as a large effect (Hopkins et al., 2009). Pearson’s product moment correlation analysis was employed to determine the relationships within and between technical parameters and TTE relative changes, as between TTE and time to attain OBLA. The following criteria was adopted to interpret the magnitude of *r*: < 0.1, trivial; 0.1–0.3, small; 0.3–0.5, moderate; 0.5–0.7, large; 0.7–0.9, very large; and 0.9–1.0, almost perfect (Hopkins et al., 2009). The null hypothesis was rejected at *P* < 0.05. Statistical analysis was performed using Sigmaplot 3.5 software (Systat Software, San Jose, CA).

Because of missing data, a linear mixed model has been used for repeated sprint NIRS results. Changes in tissues oxygenation during the RSA test were evaluated with a linear mixed model three-way repeated-measures analysis of variance (ANOVA) (time × group × sprint number). Fixed effects were time (Pre-, Post-1, Post-2), group (CON, RSN, RSH) and the sprint number (1–8), whereas subjects were considered as the random effect. Analyses were performed using R (R Core team 2017, Foundation for Statistical Computing, Vienna, Austria).

## Results

### Efficacy of the Blinding Procedure

Participants indicated not different simulated altitude of 1882 m (95% CI: 1,273–2,491 m, range, 0–3,000 m) and 1591 m (95% CI: 769–2,413 m, range, 0–4,000 m) for RSH and RSN, respectively. This indicates that the blinding process was successful and that subjects were unaware of the condition group classification.

### Training Load

Overall training load was closely matched among RSH (11,179 a.u, 95% CI: 7,074–15,284 a.u), RSN (8,366 a.u, 95% CI: 4,707–12,025 a.u) and CON: 11,678 a.u, 95% CI: 5,024–18,331 a.u) groups (*P* = 0.403). No difference in mean training load occurred between Pre- and Post-1 (RSH: 5,006 a.u, 95% CI: 2,709–7,302 a.u; RSN: 3,838 a.u, 95% CI: 1,753–5,923 a.u; CON: 5,688 a.u, 95% CI: 2,732–8,644 a.u.; *P* = 0.478), and between Post-1 and Post-2 (RSH: 6,173 a.u, 95% CI: 4,027–8,319 a.u; RSN: 4,528 a.u, 95% CI: 2,316–6,740 a.u; CON: 5,990 a.u, 95% CI: 2,131–9,849 a.u., *P* = 0.458) across groups.

### Aerobic Capacity

There was a significant interaction between time and group for TTE (*P* = 0.002, η*_*p*_*^2^ = 0.05). *Post hoc* analysis revealed that, compared to Pre, TTE increased at Post-1 (+18.3%, *P* < 0.001, d = 0.97) and Post-2 (+17.3%, *P* < 0.001, d = 0.97) in RSH, with no change in RSN and CON. Physiological responses to on-court endurance testing are summarized in Table 2.

**TABLE 2:** Performance and physiological responses to TEST before (Pre-), after the repeated sprint training in hypoxia (RSH) and normoxia (RSN) interventions or in the control group the week after (Post-1) and 3 weeks after Post-1 (Post-2).

	**RSH**			**RSN**			**Control**			**ANOVA (d)**		
	**Pre-**	**Post-1**	**Post-2**	**Pre-**	**Post-1**	**Post-2**	**Pre-**	**Post-1**	**Post-2**	**Time**	**Condition**	**Interaction**
TTE (s)	549 ± 101 [481–617]	649 ± 105* [578–720]	643 ± 95** [583–704]	582 ± 72 [534–630	582 ± 94 [519–646]	605 ± 82 [558–684]	621 ± 113 [527–716]	615 ± 90 [540–689]	604 ± 112 [523–685]	**0.008 (0.32)**	0.926 (0.12)	**0.002 (0.45)**
V̇O_2m__ax_ (mL min^–1^ kg^–1^)	57.2 ± 7.8 [52.0–62.4]	58.7 ± 7.0 [54.0–63.4]	58.5 ± 7.5 [53.7–63.3]	57.9 ± 6.0 [54.0–61.6]	57.3 ± 6.2 [53.1–61.5]	59.0 ± 7.0 [54.4–63.2]	56.2 ± 5.7 [51.4–61.0]	56.3 ± 6.1 [51.2–61.4]	57.0 ± 6.8 [51.3–62.7]	0.131 (0.13)	0.839 (0.21)	0.116 (0.11)
HR_max_ (beats min^–1^)	190 ± 8 [185–195]	191 ± 5 [188–195]	193 ± 6 [189–197]	190 ± 7 [186–195]	191 ± 8 [186–197]	191 ± 8 [185–196]	190 ± 9 [182–198]	190 ± 8 [183–197]	190 ± 8 [181–200	0.231 (0.15)	0.902 (0.16)	0.115 (0.12)
V̇E_max_ (L min^–1^)	124 ± 19 [112–137]	130 ± 17 [119–141.6]	133 ± 19 [120–145]	123 ± 11 [116–130]	124 ± 15 [114–143]	126 ± 17 [115–137]	132 ± 20 [116–149]	132 ± 24 [112–152]	128 ± 19 [117–139]	0.607 (0.13)	0.472 (0.35)	0.473 (0.24)
[La]_max_ (mMol L^–1^)	10.9 ± 4.1 [8.1–13.7]	9.7 ± 3.6 [7.3–12.1]	11.2 ± 5.2 [7.9–14.5]	8.8 ± 3.1 [6.7–10.9]	8.8 ± 2.9 [6.9–10.7]	7.2 ± 2.3 * [5.7–8.7]	9.0 ± 1.7 [7.6–10.4]	8.5 ± 2.7 [6.2–10.8]	10.3 ± 4.7 [7.9–12.7]	0.494 (0.14)	0.284 (0.46)	**0.04 (0.39)**
Time to OBLA (s)	374 ± 98 [306–439]	465 ± 118*** [384–543]	447 ± 108** [378–516]	409 ± 92 [351–468]	414 ± 111 [339–489]	451 ± 82 [399–504]	405 ± 77 [341–469]	435 ± 77 [371–499]	420 ± 82 [382–458]	**0.001 (2.56)**	0.970 (0.07)	0.097 (0.31)
Time to VT1 (s)	228 ± 114 [134–322]	288 ± 120 * [210–366]	312 ± 108* [240–384]	228 ± 48 [196–260]	234 ± 54 [198–270]	234 ± 90 [204–264]	204 ± 78 [137–268]	228 ± 60 [186–270]	234 ± 42 [159–309]	**0.019 (0.32)**	0.191 (0.56)	0.142 (0.29)
%HR_max_ at VT1	93.8 ± 2.5 [92.1–95.5]	92.2 ± 2.9 ** [90.2–94.1]	92.5 ± 2.6 [90.8–95.3]	92.1 ± 5.5 [88.4–95.8]	90.1 ± 3.5* [87.8–92.4]	89.2 ± 4.4 [86.2–92.2]	91.0 ± 4.8 [87.0–95.0]	87.8 ± 4.1 [84.4–91.2]	88.5 ± 4.8 [84.5–92.5]	**0.003 (0.44)**	0.090 (0.70)	0.401 (0.21)
Time to VT2 (s)	402 ± 102 [336–468]	480 ± 90** [420–540]	510 ± 84*** [456–564]	420 ± 96 [354–486]	408 ± 108 [336–480]	420 ± 96 [354–486]	480 ± 90 [414–546]	444 ± 72 [383–502]	474 ± 84 [402–546]	0.077 (0.26)	0.784 (0.21)	**0.004 (0.48)**
%HR_max_ at VT2	98.4 ± 1.1 [97.6–99.4]	98.2 ± 1.2 * [97.4–99.0]	98.1 ± 1.3 * [97.2–99.0]	98.8 ± 1.3 [97.7–99.9]	96.6 ± 2.0** [95.3–97.9]	96.8 ± 1.7 [95.7–97.9]	98.5 ± 1.3 [97.7–99.1]	97.2 ± 2.0 * [95.5–98.9]	97.6 ± 1.4 * [96.4–98.7]	**< 0.001 (0.58)**	0.053 (0.61)	0.167 (0.34)

Values are mean ± SD [95% CI]. Significant differences from baseline (Pre-) to the end of the intervention period (Post-1) and 3 weeks after Post-1 (Post-2). *P < 0.05, **P < 0.01, ***P < 0.001 are in bold. Statistical Analysis is based on absolute values. Cohen’s d (d) were calculated as measures of effect sizes for two-way repeated measures ANOVA. TTE, total time to exhaustion; V̇O_2m__*ax*_, maximal oxygen uptake; HR_*max*_, maximal heart rate; V̇E_*max*_, maximal ventilation; [La]_*max*_, maximal lactate concentration; OBLA, onset of blood lactate accumulation, at 4 mMol L^–1^; VT1, first ventilatory threshold; VT2, second ventilatory threshold. Note that Time to VT1 and time to VT2 only consider time of practice excluding rest between stages and specific warm-up.

Time to VT2 after the training period increased significantly only in RSH at Post-1 (+19.4%, *P* = 0.004, d = 0.06) and Post-2 (+26.9%, *P* < 0.001, d = 1.16) in reference to Pre. Concomitant changes in time to attain OBLA at Post-1 (+24.4%, *P* < 0.001, d = 0.78) and Post-2 (+19.8%, *P* = 0.003, d = 0.84) vs. Pre were observed for RSH only. Significant moderate correlation in time to attain OBLA and TTE relative changes was observed (*r* = 0.60, *P* < 0.0001), when Post-1 and Post-2 are considered vs. Pre.

### Technical Parameters

**FIGURE 2:**
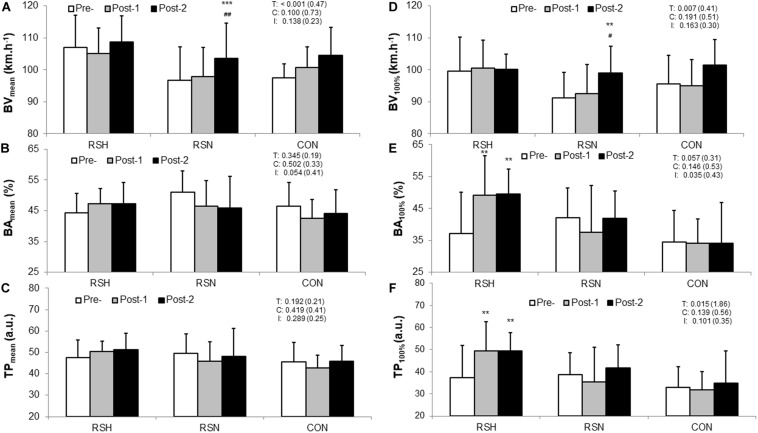
Comparison of the technical performance within and between type of intervention (RSH vs. RSN vs. CON). Representation of the overall technical performance index (TP_mean_, **C**) is calculated as the product of the ball velocity (BV_mean_, **A**) and the ball accuracy (BA_mean_, **B**) during the test to exhaustion specific to tennis. Data of the overall test are confronted to those at 100% V̇O_2m__ax_: BV_max_ **(D)**, BA_max_ **(E)**, TP_max_ **(F)**. TP is expressed in arbitrary units (a.u.). Values are Mean ± *SD*. BA is calculated as a percentage of balls in a defined zone (%). T, C, and I denote ANOVA main effects of time, group and interaction between these two factors with *P*-value and Cohen’s d (d) into brackets. ***P < 0.001, **P < 0.01 significantly different from Pre-. ^ ##^*P* < 0.01, ^#^*P* < 0.05 significantly different from Post-1.

There was a main time effect for BV_mean_ (*P* < 0.001; η*_*p*_*^2^ = 0.05) but not for TP_mean_ (*P* = 0.29, η*_*p*_*^2^ = 0.01) (Figures 2A,C).

Compared to Pre, BA_max_ at 100% V̇O_2m__ax_ increased in RSH at Post-1 (+38.2%, 95% CI: 1.0–48.4%, *P* = 0.003, d = 0.44) and Post-2 (+40.9%, 95% CI: +5.0–45.9%, *P* = 0.007, d = 0.53) (Figure 2E). There was a significant increase in BV_max_ for RSN at Post-2 (+8.7%, 95% CI: +1.5–15.9%, *P* = 0.007, d = 0.96). Increases in TP_max_ for RSH occurred at Post-1 (+41.4%, 95% CI: 0–51.6%, *P* = 0.006, d = 0.87) and Post-2 (+46.3%, 95% CI: 2.5–47.8%, *P* = 0.003, d = 1.00) compared to Pre (Figure 2F).

Significant *small* to *large* correlations were found between TP_mean_ and TTE (*r* = 0.32, *P* = 0.01), and between changes in BA_mean_ throughout the whole test and TTE (*r* = 0.53, *P* < 0.0001). TP_mean_ changes are largely correlated with those obtained in BA_mean_ (*r* = 0.89, *P* < 0.0001) when the three groups are pooled.

### Sprint-Performance Parameters

A time effect was found for RSA_best_ (*P* = 0.004; d = 0.24) and RSA_TT_ (*P* < 0.001; d = 0.21) A (Table 3). Compared with Pre-, RSH decreased RSA_best_ at Post-1 (−2.6%, 95% CI: −6.2 to +1.0%, *P* = 0.018, d = 0.63) and RSA_TT_ (−1.9%, 95% CI: −5.1 to +1.3%, *P* = 0.027, d = 0.52), and Post-2 (−5.1%, 95% CI: −8.7 to −1.5%, *P* < 0.001, d = 0.50, and −2.5%, 95% CI: −5.8 to +0.7%, 95%: *P* = 0.021, d = 0.17, for RSA_best_ and RSA_TT_, respectively). RSN also improved RSA_TT_ in the same proportion at Post-1 (*P* = 0.002) and Post-2 (*P* = 0.004) (−1.0%, 95% CI: −8.2 to +3.2%, d = 0.2), while RSA_best_ did not differ. S_dec_ did not change significantly.

**TABLE 3:** Sprint performance analysis through the best sprint (RSA_best_), total of eight sprints (RSA_TT_), and the sprint decrement (S_dec_).

	**RSH**			**RSN**			**Control**			**ANOVA (d)**		
	**Pre-**	**Post-1**	**Post-2**	**Pre-**	**Post-1**	**Post-2**	**Pre-**	**Post-1**	**Post-2**	**Time**	**Condition**	**Interaction**
RSA_best_ (s)	3.9 ± 0.1 [3.8–4.0]	3.8 ± 0.2* [3.7–**3.9]**	3.7 ± 0.2*** [3.6–**3.8]**	4.0 ± 0.2 [3.9–4.1]	3.9 ± 0.3 [3.7–4.1]	3.9 ± 0.3 [3.7–4.1]	3.9 ± 0.3 [3.6–4.2]	3.9 ± 0.2 [3.7–4.1]	3.9 ± 0.3 [3.6–4.2]	**0.004** (0.24)	0.198 (0.65)	0.150 (0.18)
RSA_TT_ (s)	31.6 ± 1.1 [30.9–32.3]	31.0 ± 1.2* [30.2–**31.8]**	30.8 ± 1.2* [30.0–**31.6]**	32.5 ± 2.0 [31.2–33.9]	32.1 ± 2.0** [30.8–**33.4]**	32.1 ± 2.0** [30.8–**33.4]**	32.3 ± 2.1 [30.5–34.1]	32.2 ± 1.9 [30.6–33.8]	32.2 ± 2.0 [30.5–33.9]	**0.006** (0.21)	0.251 (0.61)	0.585 (0.11)
Sdec (%)	2.5 ± 1.2 [1.7–3.3]	3.0 ± 0.8 [2.5–3.5]	3.4 ± 2.0 [2.1–4.7]	2.8 ± 1.1 [2.1–3.5]	2.4 ± 1.2 [1.6–3.2]	2.4 ± 1.1 [1.7–3.1]	2.8 ± 1.0 [2.0–3.6]	2.9 ± 1.4 [1.7–4.1]	2.9 ± 1.1 [2.0–3.8]	0.757 (0.11)	0.497 (0.24)	0.433 (0.31)
HR_PostRSA_ (beats min^–1^)	178 ± 8 [173–183]	177 ± 5 [173–180]	177 ± 6 [172–181]	171 ± 11 [164–179]	172 ± 8 [167–178]	171 ± 11 [163–178]	174 ± 9 [166–182]	172 ± 7. [165–178]	172 ± 10 [163–180]	0.445 (0.99)	0.207 (0.58)	0.914 (0.68)
HR_3_,_PostRSA_ (beats min^–1^)	105 ± 19 [92–117]	100 ± 15 [89–110]	101 ± 13 [92–109]	99 ± 14 [90–109]	96 ± 14 [87–106]	94 ± 13 [85–103]	103 ± 10 [94–111]	103 ± 16 [90–116]	100 ± 14 [89–112]	0.341 (0.18)	0.587 (0.31)	0.876 (0.11)
[La]_max_ (mmol L^–1^)	9.7 ± 5.3 [6.1–13.3]	8.7 ± 4.0 [6.0–11.4]	8.8 ± 2.9 [6.8–10.7]	9.1 ± 3.2 [7.0–11.2]	7.9 ± 3.4 [5.6–10.2]	7.2 ± 2.6 [5.5–8.9]	10.3 ± 2.6 [8.1–12.5]	9.5 ± 2.3 [7.6–11.4]	9.4 ± 2.4 [7.4–11.4]	0.293 (0.25)	0.568 (0.39)	0.875 (0.14)

Acute physiological responses are considered at the end of the test (HR_*PostRSA*_) and 3 min after (HR_3m__*inPostRSA*_, maximal lactate concentration ([La]_*max*_) before (Pre), the week after RSH or RSN intervention compared with control group (Post-1), and 3 weeks after Post-1 (Post-2). Data are reported as mean ± SD [95% CI]. Significant differences between Pre- and Post-1 or between Pre- and Post-2 absolute data (*P < 0.05, **P < 0.01, ***P < 0.001) are in bold. Statistical analysis is based on absolute values. Cohen’s d (d) were calculated as measures of effect size for ANOVA; T, C, and I, respectively, refer to ANOVA main effects of time, group and interaction between these two factors; with heart rate (HR), low (LF), and high (HF) frequency spectral power, LF and HF in normalized units (LFnu, HFnu), and square root of the mean of the sum of the squares of differences between adjacent normal R–R interval (RMSSD).

The maximal blood lactate concentration (values for all groups compounded) remained unchanged between Pre-(9.7 ± 2.5 mMol L^–1^), Post-1 (8.7 ± 3.8 mMol L^–1^) and Post-2 (8.2 ± 5.4 mMol L^–1^) (*P* = 0.421, d = 0.2).

### Peripheral Oxygenation

**FIGURE 3:**
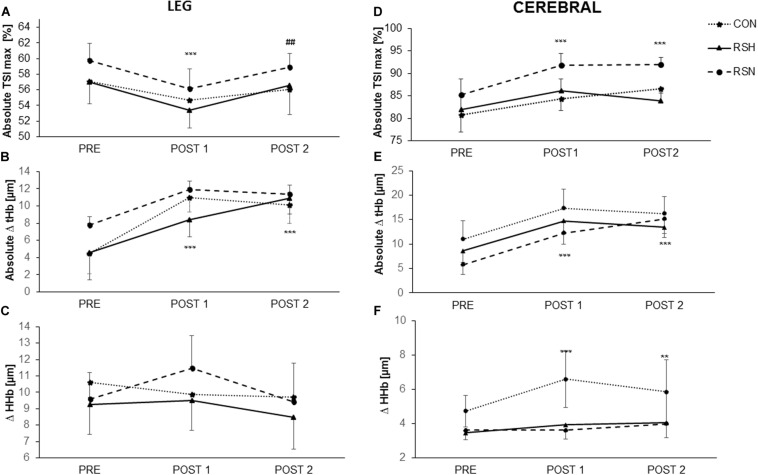
Representation of oxygenation parameters in *vastus lateralis* (leg) **(A–C)** and prefrontal cortex (cerebral) **(D–F)** during the test of repeated sprint ability. Maximal tissue saturation (%) [Absolute TSImax (%)] for leg **(A)** and cerebral **(D)**; Differences between maximum and minimum concentrations of total hemoglobin/myoglobin (Absolute Δ tHb) for leg **(B)** and cerebral **(E)**; Differences between maximum and minimum concentrations of deoxyhemoglobin (Δ HHb). For leg **(C)** and cerebral **(F)**. ***P < 0.001, **P < 0.01 significant main effect in time, different from PRE. ^##^*P* < 0.01 significant main effect in time, different from Post-1.

As indicated in Figure 3, there was an interaction between group and time for the absolute maximal TSI (TSImax). of the *vastus lateralis Post hoc* analysis indicated a lower absolute (TSImax) values at Post-1 for RSN (−3.6%, 95% CI: −5.7 to −1.5%, *P* < 0.001, d = 1.53) in reference to Pre followed by a significant increase between Post-1 and Post-2 (+2.7%, 95% CI: 0.8–4.6%, *P* = 0.003, d = 1.26).

RSH increased the absolute Δ (tHb) from Pre- to Post-1 (+82.6%, 95% CI: −94 to 248%, *P* < 0.001, d = 0.45). There was a further increase from Post-1 to Post-2 for RSH; we had a significant change from Pre- (+137%, 95% CI: −20 to 293%, *P* < 0.001, d = 0.78), differently than other groups. A main effect of time was observed when Post-2 was compared with Post-1 (*F* = 6.49, *P* = 0.01), but there was no main effect of group (Figure 3B).

A group x time interaction was found for Δ (HHb) (*F* = 4.4, *P* = 0.03) but no main effects of time and group.

### Cerebral Oxygenation

The level of cerebral oxygenation [absolute TSImax (%)] was higher in RSN with a significant increase from Pre to Post-1 (+7.8%, *P* < 0.0001), and from Pre to Post-2 (+8.1%, *P* < 0.0001). There was a significant interaction between time and group (*F* = 28.02, *P* = 0.0001) but no main effect of group.

We obtained a significant interaction for absolute Δ (thb) (*F* = 11.74, *P* = 0.0017). There was a significant effect of time (*P* < 0.001) for tHb, with *post hoc* analysis indicating significantly higher values at both Post-1 et Post-2 vs. Pre, but no effect of group existed (Figure 3E).

### HRV

There was no significant time and interaction effect for HR (*P* = 0.289, d = 0.17, *P* = 0.347, d = 0.23, respectively, in supine position; and *P* = 0.899, d = 0.06, *P* = 0.401, d = 0.25, respectively, in standing position).

No significant time and interaction effect was observed for time-domain (RMSSD) (*P* = 0.424, d = 0.42, *P* = 0.683, d = 0.36, respectively, in supine position; and *P* = 0.249, d = 0.53, *P* = 0.351, d = 0.27, respectively, in standing position). No significant time and interaction effect was found for spectral analysis (HF, LF) in either supine (*P* = 0.797, d = 0.08, *P* = 0.136, d = 0.37, for LF; *P* = 0.728, d = 0.09, *P* = 0.185, d = 0.18, for HF, respectively), or standing positions (*P* = 0.371, d = 0.45, *P* = 0.351, d = 0.11, for LF; *P* = 0.722, d = 0.13, *P* = 0.832, d = 0.05, for HF, respectively).

## Discussion

To the best of our knowledge, this is the first study examining the short-term (during the week after intervention) and prolonged effects (3 weeks after Post-1) of a hypoxic vs. normoxic specific training intervention designed for competitive tennis players. In partial agreement with our initial hypothesis, the main finding of this study was that a 12 days RSH intervention induced larger benefits for improving tennis-specific physical, physiological, and technical parameters than RSN and CON.

### Performance and Physiological Measurements

The repeated-sprint training had a positive effect on RSA parameters for both training groups with an improvement in RSA_TT_ for RSH (−1.9 and −2.5%, respectively), and RSN (−1.2% at the two time points) at Post-1 and Post-2 in reference to Pre-, yet only RSH improved RSA_best_ (−2.6 and −5.1%, respectively). These findings confirm the hypothesis that repeated sprint training ameliorates RSA, with larger benefits when conducted in hypoxia. We observed that RSH would induce beneficial adaptations mainly due to the improved blood perfusion level [absolute Δ (tHb) +82.6% at Post-1, +137% at Post-2] inducing an enhanced oxygen utilization in legs (Figure 3). The present results, however, differ from previous ones from our research group (Brechbuhl et al., 2018a), who did not report any putative effect of hypoxia on RSA parameters. Although the training protocol was similar (though without the balls hits), the RSA test consisted of 20 m sprints without any direction change. Given that the protocol entailed 16.46 m (i.e., two lengths of a tennis court baseline) sprints with 180° rotation in the middle, it could be argued that subjects developed more the neuromuscular qualities part of the sprint than maximal sprinting speed. Moreover, it has been reported in highly trained young soccer players that during a 40 m sprint, maximal speed was attained between the 30 and the 40 m distance mark (Buchheit et al., 2012). The fact that the present RSA test was more tennis-specific could explain the discrepancy in RSA results despite a similar protocol and tested population. Given that elite players on average run 3 m per shot and change four times directions during a point in official matches (Fernandez-Fernandez et al., 2009), their maximal sprinting speed will most probably never be reached. On the other hand, they will need to constantly accelerate, decelerate and reaccelerate for effective stroke production. If the participants did indeed develop deceleration and reacceleration capabilities, regardless of maximal sprinting speed, it would still be particularly relevant for improving tennis-specific performance.

In the present study, we found that RSH delayed fatigue during TEST and increased TTE (+18.3% at Post-1 and 17.3% at Post-2), whereas RSN and CON had no change. These observations confirm previous results obtained with tennis players when RSH was only compared to RSN during the first week immediately after intervention (+14.6% for RSH vs. +7.9% for RSN) (Brechbuhl et al., 2018a). In the present study, this improvement cannot be explained by a better absolute aerobic power since there was no change in V̇O_2m__ax_ or other ventilation parameters. Interestingly, the augmented time to attain OBLA suggest that toward the end of TEST, RSH spend a similar amount of time at severe exercise intensities; while the time when that particular intensity was reached occurred later. This finding is in accordance with Brechbuhl et al. (2018a), who found an increased TTE, as well as time to VT2 and time to OBLA in a similar protocol that did not incorporated actual ball hitting during training sessions. Therefore, RSH increased submaximal workload intensity for a same blood lactate concentration. A rookie professional tennis player also improved his Yo-Yo intermittent recovery test level 2 (+21% distance covered, 21 days after the training intervention) (Brechbuhl et al., 2018b). Of interest is that this beneficial effect of RSH on TTE was larger in upper-body exercise (i.e., double-poling sprint training in hypoxia) (Faiss et al., 2015). As with this study, our results showed higher improvement with balls hitting compared with previous findings based on sprints only (Brechbuhl et al., 2018a).

The variables time to attain OBLA and time of VT2 occurrence in RSH that correlated with endurance performance have been used to prescribe exercise training loads and are useful to monitor adaptation to training (Bentley et al., 2007). It has been previously demonstrated that cycling power output corresponding to OBLA improved by +7% during an incremental test after RSH only immediately after intervention (Puype et al., 2013). This is in line with the enhanced buffer capacity or upregulation of genes involved in pH control previously reported after RSH (Faiss et al., 2013b; Puype et al., 2013). This improvement in VT2 is of practical importance since it has been prioritized to be a better marker of submaximal endurance performance than V̇O_2m__ax_. Reportedly, VT2 is correlated with competitive level in male tennis player (*r* = 0.55; *P* = 0.001) (Baiget et al., 2014). According to Konig et al. (2001), high VT values could reflect the ability to tolerate high exercise intensity during tennis competitions. Additionally, our study shows that RSH was the only group to improve the time to VT1 (Table 2).

Based on our overall present results, we can speculate that physiological adaptations would likely explain improvements in time to attain OBLA, time to VT2 and TTE in the RSH group. While V̇O_2m__ax_ did not improve post-training (Figure 2), time to attain OBLA, time to VT2 and TTE increased at Post-1 (+24.4, +19.4, and +18.3%, respectively), and Post-2 (+19.8, +26.9, and +17.3%, respectively). During the RSA test, we report that RSH had beneficial adaptations concomitant with the improved blood perfusion level in their legs [absolute Δ (tHb) +82.6% at Post-1, +137% at Post-2, Figure 3] inducing an enhanced oxygen utilization. With maximal effort intensities, specific skeletal muscle adaptations (molecular level) may arise through the oxygen-sensing pathway (i.e., capillary-to-fiber ratio, fiber cross-sectional area, myoglobin content, and oxidative enzyme activity such as citrate synthase) that either do not occur in RSN or, if they do, they do so to a lesser degree (Zoll et al., 2006; Faiss et al., 2013a). As exercising in hypoxia is known to trigger a compensatory vasodilatation to match an increased oxygen demand at the muscular level (Casey and Joyner, 2012), hypoxic and maximal effort intensity are combined to increase the training stimulus. However, unchanged V̇O_2m__ax_ values may be explained by the unchanged cardiac output which could be measured in future study.

### Fatigue and HRV

An interesting and novel finding of the present study was the lack of differences between groups and time points in markers of autonomic fatigue. This observation is not in line with previous responses to altitude training (“Living high-training low” paradigm) that remain complex since hypoxia and training stresses are combined (Schmitt et al., 2006). In hypoxia, the reduced inspired pressure of oxygen represents an additional stress, which has been shown to alter the autonomous regulation of the nervous system (Schmitt et al., 2006). Responses in HRV are subsequently with a combination of increased sympathetic and decreased parasympathetic nervous activities (Schmitt et al., 2006). A recent study showed also that HRV returned to basal values the day following a RSH session, which suggests that the exercise performed did not elicit long-lasting effects in autonomic modulation (Valenzuela et al., 2019). It also confirms previous psychophysiological responses to repeated sprints training. Despite higher hypoxia-induced physiological and perceptual strain during the first session, perceptual responses improved thereafter in RSH so as not to differ from RSN (Brocherie et al., 2017b). This already indicated an effective acclimation and tolerance to this innovative training.

### Technical Performance Measurements

The TP_max_ enhancement of the RSH group (Figure 2F) during TEST were mainly related to improved BA_max_ (Figure 2E) and cannot be explained by a lack of involvement in ball hitting in favor of lower energy consumption. We did not find significant differences in BV_mean_ (Figure 2A) between RSH and the other two groups from Pre- to Post-tests as it was the case for BV_max_ at 100% V̇O_2m__ax_ (Figure 2D). Moreover, the level of engagement in strokes is also confirmed by the exhaustion criteria (i.e., VE_max_, HR_max_) that were similar in both groups (Table 2).

BA_max_ significantly increased in RSH, whereas it decreased slightly in RSN and did not differ in CON (Figure 2E). While a potential learning effect, due to unchanged data obtained by CON, cannot be ruled out it seems that fatigue resistance was improved for RSH, which may potentially be transferable to technical performance gains. However, few investigations conducted on cognitive skills (e.g., visual search and decision making) in hypobaric or normobaric hypoxic conditions have demonstrated their acute effects on attention, perception, executive functioning and short-term memory (Mcmorris et al., 2017). Our study does not confirm potential effect on cerebral oxygenation kinetics following RSH intervention to explain why this training group had higher accuracy scores near exhaustion (Brechbuhl et al., 2018a). For practical reasons NIRS evaluation was only conducted during RSA test so that evaluation of technical performance during TEST would not be disturbed.

### Practical Applications

With the modern tennis game becoming increasingly dynamic and tournament schedules more demanding, adapted strategy to improve or maintain physical fitness are needed (Reid and Schneiker, 2008). In this context, additional adaptations by using upper body (e.g., with ball hitting) during RSH with its positive influence on RSA and muscle blood perfusion (Faiss et al., 2015) appear more suitable than extending the length of protocol. RSH is an effective short-term intervention to develop and maintain tennis-specific capabilities to maximize fitness when players are involved in a series of 3–4 consecutive tournaments. We propose that this intervention can be programmed 2–3 times per season in order to develop or maintain the athletes’ aerobic capacity and BA. Indeed, five sessions of RSH spread over 12 days could trigger adaptations beneficial for both sprinting and tennis-specific performance with no additional fatigue.

### Limitations and Perspectives

Despite being tested in ecological situation, players were wearing a portable analyzer (additional weight of ∼0.7 kg), while the mask might have also affected vision. However, it is important to note that all players during both Pre- and Post- Tests sessions wore this equipment, so any potential negative effect (i.e., earlier fatigue due to extra weight to carry or impaired accuracy due to perturbations in vision) would have been similar in all situations. We acknowledge that it is important to use valid and reliable test procedures, also including tests of RSA. Despite the large number of tests of RSA available in the literature, no gold standard procedure existing for the purpose of testing tennis players on this fitness component. Tennis Australia recommends a test including 10 × 20 m straight-line sprints with 20 s rest (Fernandez-Fernandez et al., 2014). A more tennis-specific test, including 10 × ∼20 m shuttle sprints was also implemented with tennis players (Fernandez-Fernandez et al., 2012). When testing RSA it is crucial to avoid occurrence of pacing and so short and tennis specific drill are important characteristics of the test selected. Importantly, we also familiarized our participants with this testing procedure before pre-tests to exclude any learning effects.

## Conclusion

Five RSH sessions over 12 days is an efficient strategy for causing larger immediate improvements in tennis-specific anaerobic, aerobic, and technical performance compared to RSN and a control group. Practically, RSH also benefited on-court technical performance, particularly the ball accuracy near exhaustion (100% V̇O_2m__ax_). Improved repeated-sprint ability was accompanied by increases in marker of blood perfusion. In addition, markers of autonomic modulation remained largely unaffected under the conditions of the present study.

Unique was also the fact that most of these aforementioned gains are maintained at least 4 weeks in the RSH group only.

## Data Availability Statement

The raw data supporting the conclusions of this article will be made available by the authors, without undue reservation.

## Ethics Statement

The studies involving human participants were reviewed and approved by French National Conference of Research Ethics Committees—Ile de France 6, No. 77-17. The patients/participants provided their written informed consent to participate in this study.

## Author Contributions

CB, LS, GM, and BM conceived and designed the experiments. CB, FB, LS, TB, and BM performed the experiments. CB, LS, GM, TB, and SW analyzed the data. CB, LS, TB, SW, and FB contributed reagents, materials, and analysis tools. CB and LS wrote the manuscript. CB, OG, GM, LS, and FB critical revision of the manuscript for important intellectual content. All authors contributed to the article and approved the submitted version.

## Conflict of Interest

The authors declare that the research was conducted in the absence of any commercial or financial relationships that could be construed as a potential conflict of interest.
